# Insect-Derived Anti-Aging Bioresources: Current Status, Bottlenecks, and Future Directions

**DOI:** 10.3390/insects17080847

**Published:** 2026-08-14

**Authors:** Minghui Zhao, Weina Wang, Hongyu Lai, Lixin Zhou, Qi Liao, Jinxin Liu, Hongning Liu, Miao Ouyang, Gang Ren, Zishu Dong

**Affiliations:** 1 Advanced Research Institute, Jiangxi University of Chinese Medicine, Nanchang 330004, China; zhaominghui@jxutcm.edu.cn (M.Z.); wangweina@jxutcm.edu.cn (W.W.); laihongyu@jxutcm.edu.cn (H.L.); zhoulixin@jxutcm.edu.cn (L.Z.); liujinxin@jxutcm.edu.cn (J.L.);; 2 Research Center for Differention and Development of TCM Basic Theory, Jiangxi University of Chinese Medicine, Nanchang 330004, China; 3 Research Center of Natural Resources of Chinese Medicine Materials and Ethnic Medicine, Jiangxi University of Chinese Medicine, Nanchang 330004, China; 4 Student Affairs Office, Jiangxi University of Chinese Medicine, Nanchang 330004, China

**Keywords:** insects, anti-aging, bioactive compounds, preparation methods, mechanisms of action

## Abstract

As the global population ages rapidly, demand for safe and effective strategies to promote healthy aging grows markedly. Natural bioactive compounds, with favorable biosafety profiles and multi-target regulatory properties, have become a core focus in developing anti-aging functional foods and nutraceuticals. Amid the search for novel sustainable bioresources, insects emerge as promising anti-aging candidates for their rich species diversity, scalable production, low environmental footprint, and abundant unique bioactive components such as functional proteins and bioactive peptides. Based on bibliometric analysis of 500 eligible publications spanning two decades, this review systematically identifies 32 anti-aging insect species across seven orders, classifies their bioactive components into five major categories, summarizes green extraction technologies, and evaluates their in vitro and in vivo anti-aging activities centered on oxidative stress and inflammatory regulation, while outlining the field’s trajectory from basic mechanistic research to functional application. It further highlights core challenges including fragmented research frameworks and insufficient robust in vivo validation. Finally, it recommends integrating established food science and medical methodologies with emerging technologies such as omics, artificial intelligence, and advanced delivery systems to advance future research paradigms. These efforts could provide a strong theoretical foundation for the efficient and sustainable use of insect resources in anti-aging applications.

## Simple Summary

In recent years, with deepening global population aging, demand for effective anti-aging regimens has grown rapidly. At present, most anti-aging interventions depend on plant extracts and synthetic compounds. Plant materials mitigate aging manifestations but face supply and cultivation constraints, while synthetic agents carry long-term safety risks, which has drawn researchers’ attention. Therefore, developing sustainable novel anti-aging raw materials becomes an important subject. Compared with plant and synthetic materials, insects, which contain abundant bioactive ingredients, are a greener and viable alternative resource. This review paper systematically summarizes 32 anti-aging insect species across seven orders, classifies their bioactive components into five major groups, analyzes key obstacles restricting their utilization, and provides a theoretical basis for the exploitation of insect-derived anti-aging resources.

## 1. Introduction

The rapid aging of the global population, coupled with growing public health awareness, is fueling the rapid expansion of the anti-aging market [1]. Currently, most natural anti-aging interventions focus on nutritional health products, dietary supplements, functional foods, and natural antioxidants. Market forecasts predict the global anti-aging market will maintain sustained growth [2], with the supplement segment occupying a dominant market share, highlighting the significant commercial potential of natural bioactive compounds for promoting healthy aging. Benefiting from favorable safety profiles, minimal side effects, and multi-target regulatory properties, natural bioactive agents have become a central research focus for innovative anti-aging strategies [3]. At present, research and development largely centers on plant-based resources. While plant extracts can deliver anti-aging benefits by influencing processes such as oxidative stress and chronic inflammation [4], limited supplies and long cultivation periods [5] severely hamper their ability to meet rising market demand. As a result, resource scarcity is now a major bottleneck constraining the sustainable development of the anti-aging industry. This situation underscores the urgent need to identify sustainable and readily available alternative natural resources. As the most species-diverse and biomass-abundant organisms on Earth, insects possess several advantageous characteristics, including rapid reproductive cycles [6], low carbon footprints [7], low breeding costs [8], and high resource conversion efficiency [7]. Owing to these advantages, insects have attracted increasing attention as promising resources for the development of natural anti-aging products. In addition, numerous insect species and their tissues are rich in a wide range of bioactive compounds, further highlighting their potential for anti-aging applications. For example, royal jelly, which is enriched with fatty acids, proteins, and other bioactive components, has been shown to improve antioxidant status and delay cellular senescence [9]. Similarly, polysaccharides extracted from *Periplaneta americana* Linnaeus can regulate immune function and suppress inflammatory responses, thereby providing novel targets for anti-aging research [10]. Moreover, insect resources are not constrained by seasonal or geographical limitations and can be continuously supplied through industrial-scale breeding systems. This stable production capacity can effectively alleviate the shortage of natural anti-aging raw materials [11]. Therefore, the systematic exploration of insect-derived bioactive substances may not only expand the resource base for natural anti-aging agents but also provide new perspectives for the sustainable development of the anti-aging industry.

The use of insects in anti-aging interventions is not a new area of research; it has long been rooted in traditional practices and empirical knowledge. Classical Chinese medical texts describe *Ophiocordyceps sinensis* (Berk.) Sung et al. as a remedy that “nourishes deficiencies and enhances vital energy,” underscoring its historical role in delaying aging and boosting immune function for thousands of years [12]. Similarly, silkworm pupae have been traditionally used as both food and medicine in dietary therapies throughout East Asian countries, including Japan and South Korea, to combat physical weakness and slow aging [13]. These widespread, cross-cultural applications offer valuable empirical evidence and a strong historical basis for modern research on insect-based anti-aging strategies. With rapid advances in omics technologies and molecular biology, research on insect-derived anti-aging substances has evolved from traditional empirical use to systematic scientific validation. Growing evidence has illuminated the molecular mechanisms behind the anti-aging effects of insect-derived bioactive compounds. For instance, extracts from *Tenebrio molitor* Linnaeus have been shown to delay cellular aging by upregulating proteins such as SIRT1 [14]. In addition, hydrolysates of *Schistocerca gregaria* Forsskål possess free radical scavenging activity similar to common plant-derived antioxidants [15]. Together, these findings provide strong scientific support for the effectiveness of insect-based anti-aging strategies and lay a solid foundation for targeted compound screening and improved preparation technologies.

Despite notable progress in insect-based anti-aging research, significant gaps remain in both scientific understanding and industrial application. Insects account for roughly 46% of all known species on Earth [16]. However, a systematic review by the Food and Agriculture Organization of the United Nations (FAO) and Wageningen University found that only about 2000 insect species are considered edible worldwide [17]. Strikingly, less than 0.01% of all insect species have been thoroughly studied or used for medicinal purposes [18], reflecting the underutilization of this vast resource. Furthermore, current research is fragmented and lacks systematic criteria for selecting key insect species, which hinders the coordinated application of findings. Existing studies on the anti-aging mechanisms of insect-derived bioactive compounds also remain limited, and a comprehensive research framework that addresses multiple biological targets and signaling pathways has yet to be established. Furthermore, a significant disconnect between laboratory research and commercial production severely limits the industrial transformation and practical application of related research achievements. In addition to these constraints, unresolved issues such as unclear structure–activity relationships, insufficient in vivo validation, and the absence of standardized quality control systems further hinder the industrial transformation and large-scale application of insect-derived anti-aging resources. In this context, the present study employs bibliometric visualization methods to systematically map the overall research landscape of insect-based anti-aging fields. This research synthesizes existing data on anti-aging insect species and their bioactive components, delves into their underlying anti-aging molecular mechanisms, assesses their industrial application potential, and proposes targeted optimization and development strategies. The study aims to provide a robust theoretical foundation and practical guidance for the further exploration of insect-derived anti-aging resources and the effective industrial translation of relevant scientific research achievements.

## 2. Visual Analysis of Anti-Aging Research in Insects

To systematically clarify the current state and evolving trends in insect-based anti-aging research, this study conducted a bibliometric visual analysis of the relevant literature published between January 2005 and December 2025, using the Web of Science, Scopus, and PubMed databases. The study aimed to identify core research hotspots, developmental trends, and emerging topics in this field. The analysis was performed with CiteSpace 6.4 R1. Time slicing covered 1 January 2005 to 31 December 2025, with one-year slices. Within each slice, links were built based on cosine intensity. The screening criterion used the g-index with k = 25, and the pruning strategy was Pathfinder plus Pruning Sliced Networks. Node types included Author, Keyword, Institution, Country, Reference, Cited Author, and Cited Journal, with the inclusion criterion defined by g-index (k = 25). Inclusion criteria were English-language articles only, limited to research articles and reviews, published between January 2005 and December 2025. Retrieval was defined as follows: TS = ((insect* OR arthropod*) AND (“anti-aging” OR “lifespan extension” OR “senescence delay”) AND (polyphenol* OR “antimicrobial peptide” OR chitin) AND (autophagy OR “oxidative stress” OR telomerase)) NOT TS = (“animal*” OR “plant*”). Exclusion criteria were letters, news items, conference abstracts/papers, editorial materials, and book chapters; unpublished grey literature; retracted publications and duplicates; and documents lacking information essential for bibliometric analysis. After relevance screening and deduplication, 500 eligible publications were obtained in total, comprising 249 from Scopus, 246 from Web of Science, and 5 from PubMed. These publications involved 2097 authors, 949 institutions, and 78 countries/regions.

**Figure 1:**
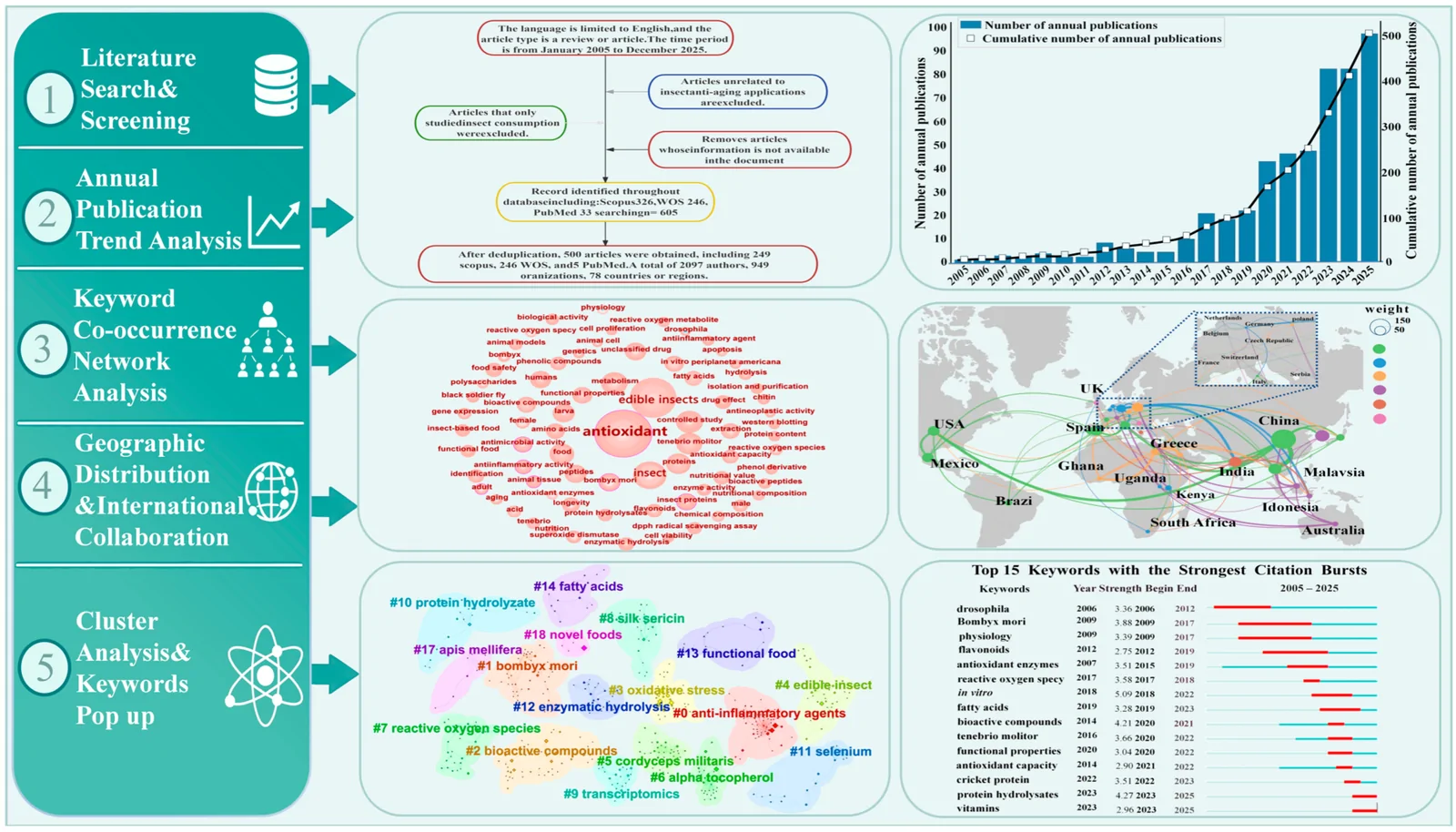
Bibliometric analysis of research trends in insect anti-aging studies [19]. Global bibliometric distribution analysis further shows that China occupies a core research position in this field and has established close academic cooperation with the United States, Mexico, Brazil, Spain, and other countries.

Based on the final dataset, statistical analysis of annual publication output showed that the field has developed rapidly. In 2005, only two relevant papers were published. After 2016, the field entered a rapid growth phase, with annual publications exceeding 20 in 2017 and peaking at 81 in 2022. Growth slowed between 2020 and 2022, likely due to experimental and publication delays caused by the COVID19 pandemic. However, the trend quickly rebounded, with 91 papers published in 2025. Overall, cumulative publications have increased exponentially, highlighting insect-based anti-aging research as a rising hotspot at the intersection of food science, nutrition, and geriatric medicine (Figure 1).

High-frequency keywords (aging, longevity, metabolism, oxidative stress, antioxidant activity, bioactive compounds, protein hydrolysate, *T. molitor*, *Bombyx mori* Linnaeus) reveal the core focus of current research: insect-derived antioxidant peptides and bioactive compounds delay aging by regulating oxidative stress and improving metabolic pathways. Among the investigated insect resources, *T. molitor* and *B. mori* are the most representative insect species. Their protein hydrolysates and intrinsic antioxidant components have been widely adopted for in vitro and in vivo validation of their anti-aging mechanisms.

Keyword emergence and cluster analyses clearly reveal the evolutionary trajectory of research content in this field. Over the past two decades, insect-based anti-aging research has gradually expanded its research scope from the analysis of basic nutritional components to the multidimensional exploration of biological activity mechanisms. Early investigations in this field were primarily centered on the screening of model organisms and the elucidation of fundamental anti-aging mechanisms, whereas subsequent research phases increasingly emphasized the identification of bioactive compounds and the evaluation of their functional properties. In recent years, research topics such as antioxidant activity, protein hydrolysates, and functional foods have emerged as prominent keywords, reflecting the coordinated advancement of both fundamental and applied research. Cluster analysis further categorized current research hotspots into several major thematic domains, including anti-inflammatory responses, oxidative stress, α-tocopherol, protein hydrolysates, and functional foods. Collectively, the evolution of research priorities demonstrates a clear transition from preliminary model establishment and mechanistic exploration toward comprehensive mechanistic elucidation and industrial translation. Notably, during the past five years, research emphasis has progressively shifted from basic mechanistic studies to functional evaluation and product-oriented development. Furthermore, the increasing prominence of protein hydrolysates and vitamin-related research suggests that the targeted delivery and precision application of insect-derived bioactive peptides are likely to emerge as key frontier directions in anti-aging research over the next 2–3 years.

## 3. Bioactive Anti-Aging Components of Insects and Corresponding Extraction Technologies

**Figure 2:**
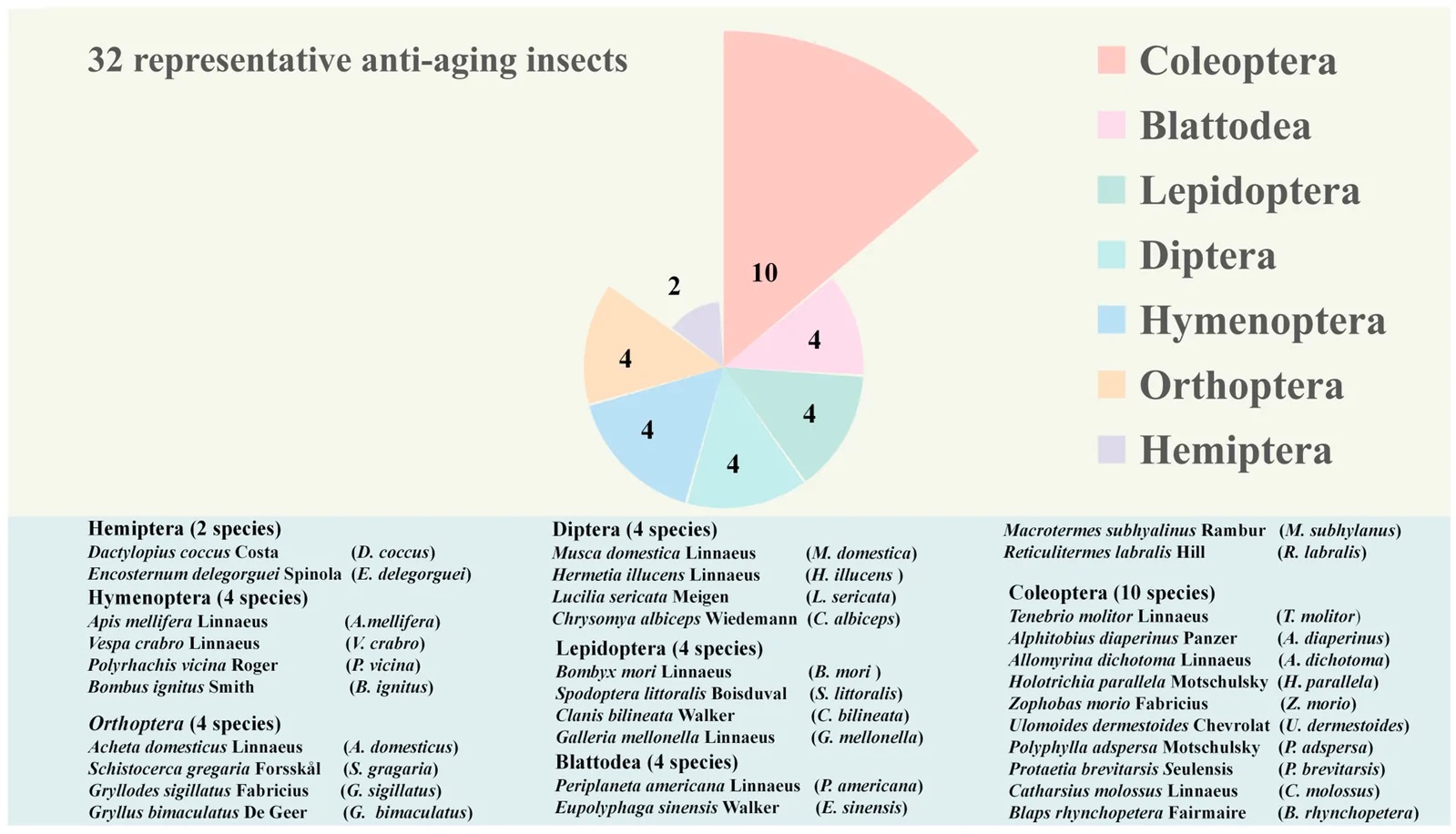
The classification of insect species with clear anti-aging potential screened through the literature in seven orders.

As one of the most diverse and resource-rich biological groups on Earth, insects contain a wide array of substances with potential anti-aging properties. However, the composition and distribution of these bioactive compounds vary significantly among insect species. Consequently, targeted extraction methods and optimized processing technologies are necessary to efficiently isolate and utilize specific bioactive components (Figure 2).

### 3.1. Anti-Aging Insect Species

Anti-aging insects refer to insect species that are rich in bioactive compounds, including proteins and peptides [20], lipids [21], polyphenols [22], polysaccharides [23], and trace elements [24]. These bioactive constituents can delay aging and promote a healthy lifespan through multiple regulatory mechanisms, thereby demonstrating considerable potential for applications in functional foods [25], skincare products [26], and dietary supplements [27]. To date, insects belonging to seven orders, namely, Coleoptera, Orthoptera, Hymenoptera, Lepidoptera, Blattodea, Hemiptera, and Diptera, have been scientifically reported to exhibit significant anti-aging potential, with 32 species having been explicitly identified and investigated; refer to Figure 2 and Supplementary Table S1. Well-documented insect species with solid anti-aging evidence include *B. mori*, *Apis mellifera* Linnaeus, *Hermetia illucens* Linnaeus, *T. molitor*, *Acheta domesticus* Linnaeus, *Clanis bilineata* Tsingtauica, and *Catharsius molossus* Linnaeus [13,25,28,29]. China has a long-standing tradition of utilizing insect resources for health maintenance and disease intervention. Multiple traditional medicinal and edible insects, such as silkworm pupae, *A. mellifera* larvae, and *T. molitor*, have been validated for anti-aging efficacy in modern pharmacological studies. For Coleoptera, *T. molitor* protein concentrates exert protective effects in aging mouse models by enhancing antioxidant capacity, alleviating inflammatory responses, and improving intestinal health [25]. For Orthoptera, protein hydrolysates derived from *Gryllus bimaculatus* De Geer produced bioactive peptides with prominent antioxidant activities [30]. For Hymenoptera, *A. mellifera* has well-defined anti-aging mechanisms, endowing it with great potential for translational research and industrial transformation [28]. As a typical Lepidoptera insect, *B. mori* serves as an excellent source of antioxidant peptides [20]. As an emerging resource of Diptera, *H. illucens* contains unique metabolites that provide novel directions for anti-aging research [31].

### 3.2. Anti-Aging Active Ingredients

Insect-derived active ingredients with anti-aging potential can be classified into five major categories: proteins, lipids, polyphenols, polysaccharides, and trace elements (Table 1).

#### 3.2.1. Proteins and Peptides

Insects possess a total protein content ranging from 35% to 60% of their dry weight and 10% to 25% of their fresh weight, which is considerably higher than that found in traditional grains and legumes. Specifically, Orthoptera insects display particularly high protein levels, with their protein content peaking at approximately 60% on a dry weight basis [46]. These insect proteins contain all essential amino acids required for human metabolism and nutrition, featuring high digestibility and absorbability. Beyond basal nutritional support, enzymatic hydrolysis acts as a critical step to unlock and amplify the direct anti-aging bioactivity of insect proteins: intact insect proteins already exhibit direct anti-senescence capacity, as demonstrated by the prominent inhibitory effect of major royal jelly proteins on senescence in human embryonic lung fibroblasts (HFL-I) [47]. Insect-derived complete proteins can be degraded into protein hydrolysates via enzymatic hydrolysis, and the antioxidant, anti-inflammatory, and anti-aging bioactivities of such hydrolysates have been widely validated in multiple insect species. At the cellular level, *B. mori* sericin protein hydrolysate alleviated cellular senescence in human dermal fibroblasts. It reduced intracellular reactive oxygen species (ROS) accumulation, upregulated the expression of superoxide dismutase 2 (SOD2) and catalase (CAT), mitigated oxidative damage, and restored extracellular matrix homeostasis [36]. Similarly, *H*. *illucens* alleviated cellular senescence in human dermal fibroblasts. It reduced intracellular reactive ROS accumulation, upregulated the expression of SOD2 and CAT, mitigated oxidative damage, and restored extracellular matrix homeostasis [48]. Studies further verified that aqueous protein hydrolysates from *T*. *molitor* and *Protaetia brevitarsis seulensis* Kolbe alleviated intestinal inflammation and improved cognitive performance in aged mice through anti-inflammatory modulation [14].

Insect-derived bioactive peptides act as the core functional units driving anti-aging effects and are classified into two primary categories: antioxidant peptides and immunomodulatory peptides. Antioxidant peptides directly counteract age-related oxidative damage by scavenging excess reactive species and blocking oxidative cascade reactions. Two representative fragments, SWFVTPF and NDVLFF, identified from B. mori pupal hydrolysates, exhibit robust comprehensive antioxidant capacity that counteracts macromolecular damage accumulated during aging [20]. The anti-aging activity profile of peptide products can be optimized via enzyme selection: hydrolysates prepared with Alcalase and Prolyve exert stronger free radical scavenging efficacy due to their abundant low-molecular-weight peptides, while Flavourzyme-generated hydrolysates show superior iron-reducing capacity [20]. As a core subtype of immunomodulatory peptides, antimicrobial peptides primarily exert their anti-aging effects through multiple mechanisms, including regulation of gut microbiota homeostasis and suppression of chronic inflammation [49]. In LPS-induced macrophage experiments, cecropin antimicrobial peptides specifically bound to LPS, thereby markedly downregulating the expression of pro-inflammatory factors and related genes and alleviating inflammatory damage [50]. Currently, anti-aging research on insect proteins mostly focuses on activity validation of enzymatic hydrolysates, and systematic studies on their structure–activity relationships and in vivo action mechanisms still need to be further expanded.

#### 3.2.2. Lipids

Lipid metabolic disorders are closely associated with fundamental mechanisms of aging, particularly oxidative stress and chronic inflammation and therefore represent important therapeutic targets for the prevention and treatment of aging and age-related diseases [51]. In insects, lipids are the second most abundant class of functional nutrients after proteins and can be broadly categorized into fatty acids, wax esters, and phospholipids. Among these components, fatty acids constitute the major bioactive fraction, with unsaturated fatty acids contributing predominantly to anti-aging activity [27]. Specifically, distinct fatty acid profiles endow different insect lipids with differentiated anti-aging action pathways: lipids from *H. illucens* larvae, enriched in lauric acid, oleic acid and linoleic acid, focus on cutaneous aging by repairing the epidermal barrier and mitigating local oxidative and inflammatory damage [26]; in contrast, lipids from *B. mori,* which are abundant in α-linolenic acid and have an n-6/n-3 ratio close to the optimal nutritional range, exert systemic anti-aging effects by maintaining whole-body lipid homeostasis [21,51]. Beyond fatty acids, specialized lipid components further expand the application boundaries of insect lipids: the wax ester fraction MUD1 from *A. mellifera* beeswax by-products protects human dermal fibroblasts from oxidant-induced damage [52], and insect-derived polyunsaturated fatty acid-phospholipid complexes combine enhanced antioxidant activity with favorable emulsifying properties, showing great potential for anti-aging dietary supplements and nutritional fortifiers [27].

#### 3.2.3. Polyphenols

Insects are rich in diverse bioactive polyphenols, mainly encompassing 4-hydroxybenzoic acid, p-hydroxycinnamic acid, ferulic acid, eugenic acid, and flavonoids [43]. These polyphenols are primarily acquired via food chain accumulation and distributed across the insect cuticle, defensive glands and gut, performing dual biological functions in organismal defense and anti-aging regulation [41]. Polyphenolic compounds derived from insects possess significant antioxidant activity and exert anti-aging effects by scavenging excess free radicals in the body and alleviating oxidative stress-induced cellular damage. The polyphenolic components of the ethanol extract of *Holotrichia parallela* Motschulsky exhibit antioxidant activity [22]. Beyond systemic antioxidant protection, insect polyphenols further exert targeted intervention effects on tissue-specific aging phenotypes, with skin photoaging improvement being the most well-validated application direction. Tpolyphenols extracted from *A. domesticus* extracts suppress the activities of collagenase and elastase to attenuate collagen degradation. These compounds also upregulate the TGF-β1 signaling pathway to facilitate collagen synthesis. Such dual regulatory effects effectively alleviate photoaging and diminish wrinkle formation, endowing insect polyphenols with great application potential in anti-aging cosmetic development [53].

#### 3.2.4. Polysaccharides

Insect polysaccharides fall into two major classes: structural polysaccharides and functional polysaccharides. Functional polysaccharides, typically exemplified by the CP70 polysaccharide from *Paecilomyces cicadae* (Miq.) Samson, are freely distributed in insect hemolymph, body cavities, and secretions and exhibit diverse biological activities. The CP70 polysaccharide isolated from *Paecilomyces cicadae* upregulated the mRNA levels of catalase, superoxide dismutase 1, and nucleic acid oxidation damage repair enzymes in *Drosophila melanogaster* Meigen. Such modulation enhanced the activities of catalase and glutathione peroxidase, decreased the content of malondialdehyde, a key marker of lipid peroxidation, and ultimately strengthened cellular antioxidant defense. These beneficial effects effectively prolonged the healthy lifespan of fruit flies [44]. Glycosaminoglycans (GAGs) extracted from *C. molossus* mitigate oxidative damage and inhibit inflammatory responses in aged rat models. The multi-target regulatory effects confer cardioprotective and antithrombotic functions, thereby delaying multi-organ aging progression [23]. Chitin and its deacetylated product chitosan are typical structural polysaccharides of insect origin. Both biomaterials are polymerized by N-acetyl-D-glucosamine through β-1,4 glycosidic linkages [54], and insects constitute the predominant renewable source of terrestrial chitin. Chitosan derived from *C. bilineata* larvae alleviated oxidative stress in D-galactose-induced aging mice [45]. Collectively, insect polysaccharides exert anti-aging effects through multiple complementary pathways, highlighting their considerable development potential in anti-aging functional products.

#### 3.2.5. Trace Elements

Trace elements are essential nutrients for sustaining human physiological homeostasis. They participate in and regulate core biological processes, including metabolism, immune modulation, hormone synthesis, and signal transduction. The disruption of trace element homeostasis closely correlates with elevated oxidative stress, persistent chronic inflammation, and accelerated cellular senescence [55]. Insects contain abundant macroelements and trace elements, mainly covering iron, zinc, potassium, sodium, calcium, phosphorus, magnesium, manganese, and copper. Among these mineral components, zinc, copper, and selenium serve as the key functional substances mediating the anti-aging bioactivities of insects [56]. Studies have confirmed that the larvae of the giant blowfly *Chrysomya megacephala* Fabricius, *T. molitor*, and other insects have short growth cycles and high reproductive efficiency [57]. These insect species efficiently utilize inorganic aquaculture waste and biotransform inorganic selenium into organic selenium. This bioconversion process markedly improves selenium bioavailability and enhances its anti-aging capacity, suggesting that insects can serve as a high-quality and sustainable trace element source for the development of anti-aging functional substances.

### 3.3. Extraction Processes for Anti-Aging Active Ingredients

**Figure 3:**
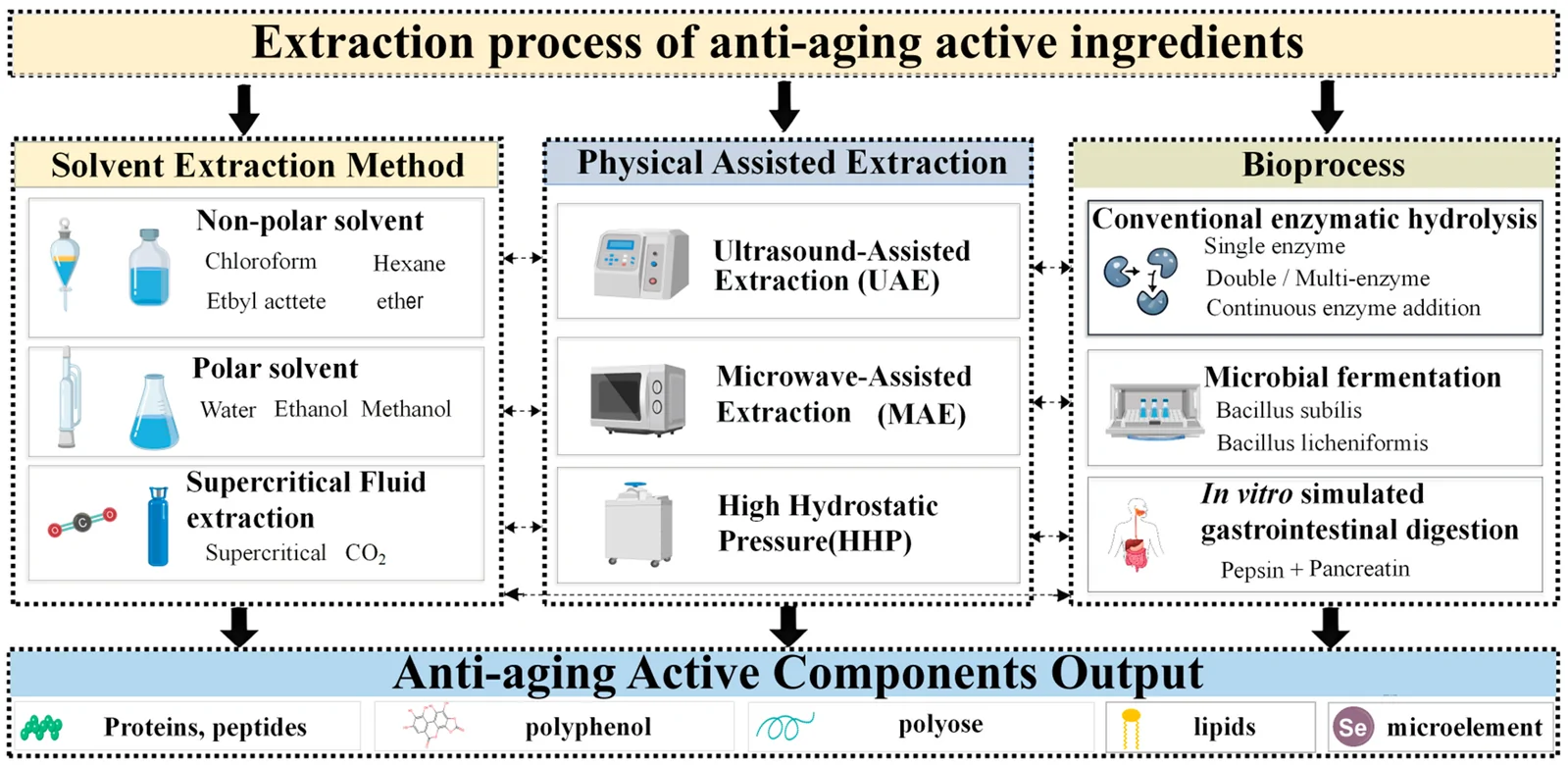
Preparation and extraction flow of insect-derived anti-aging ingredients.

Based on the physicochemical characteristics of target active ingredients, current extraction techniques are primarily divided into three categories: physical-assisted extraction, solvent extraction, and bioprocessing (Figure 3).

#### 3.3.1. Physically Assisted Extraction

Physically assisted extraction disrupts insect cellular structures, enhances mass transfer efficiency, and promotes the release of target bioactive substances via external physical fields, mainly including ultrasound, microwaves, and high hydrostatic pressure. Each technique has distinct action mechanisms and application advantages. Among these methods, ultrasonic-assisted extraction (UAE) is especially useful for removing heat-sensitive materials such as proteins, peptides, polysaccharides, and polyphenols because it uses high-frequency vibrations and cavitation effects to break down cell structures [58]. This feature makes it the preferred cost-effective strategy for heat-sensitive anti-aging bioactives, including proteins, peptides, polysaccharides, and polyphenols. The foundation of microwave-assisted extraction (MAE) is the quick and targeted heating of polar molecules using microwave energy. This technique is optimally suited for heat-stable, polar small-molecule anti-aging components such as polyphenols and phenolic derivatives, which are core active ingredients for antioxidant and anti-skin-aging applications [43]. High-pressure-assisted extraction (HHP-E) uses ultra-high pressures between 100 and 600 MPa to break down cell walls. Notwithstanding their extraction efficacy, each method has inherent scale-up constraints: prolonged ultrasound may cause structural degradation of macromolecular actives [59]; microwave-induced localized heating carries thermal damage risks for heat-sensitive components [60]; and high-pressure processing requires high upfront capital investment and strict safety management [61]. Accumulated experimental evidence verifies the extraction efficacy of these methods for insect anti-aging components. This non-thermal processing characteristic makes it particularly suitable for pressure-stable but heat-labile anti-aging components, including bioactive polysaccharides and unsaturated fatty acid-rich insect oils. Applying ultrasonic-assisted extraction to proteins from *T. molitor* larvae under alkaline conditions not only greatly boosts protein yield but also decreases protein particle size, encourages the unfolding of protein spatial structures, and improves the exposure of functional amino acid residues [59]. Additionally, by exposing more proteolytic cleavage sites and unfolding protein structures, ultrasonic pretreatment greatly increases the hydrolytic effectiveness of later proteases like Alcalase. Consequently, the resultant hydrolysates show higher iron-chelating activity and improved DPPH radical scavenging capacity [62]. Consistent with this, phenolic extracts from *T. molitor* obtained via MAE exhibit higher total phenolic content and stronger antioxidant activity compared with those from conventional extraction methods [63]. A polysaccharide yield of 14.89 ± 0.68% was obtained during the extraction of polysaccharides from Cordyceps militaris under optimized HHP-E conditions [64]. Additionally, during the extraction of insect oil from *A. domesticus* and *T. molitor*, this method significantly increased the contents of certain fatty acids and improved antioxidant activity [65]. In summary, the optimal extraction strategy depends on the properties of the target anti-aging compound and downstream product positioning: for protein/peptide/polysaccharide ingredients targeting mass-market functional foods, UAE provides the best balance of activity preservation and cost efficiency [66]; for polyphenol-based raw materials for skincare and general dietary supplements, MAE offers superior throughput and cost performance [64]; for high-value, heat-sensitive active oils and polysaccharides in premium applications, HHP-E is the preferred option despite higher costs [65]. Mechanistically, all physical-assisted extraction methods enhance efficiency via cell wall disruption and intensified mass transfer, and their commonly used aqueous systems also confer environmental benefits, but their industrial scalability is ultimately constrained by the match between their technical characteristics and target product value propositions.

#### 3.3.2. Solvent Extraction

One of the most well-known and popular methods for separating natural compounds is solvent extraction. This approach, which is based on the idea that “like dissolves like,” provides a straightforward operational procedure, adaptable parameter adjustment, and wide application from laboratory study to industrial-scale production [67]. Polar solvents (ethanol, water, and their mixed systems) are primarily applied to extract water-soluble anti-aging components such as bioactive peptides, polyphenols, and polysaccharides, while nonpolar solvents (n-hexane, chloroform) are used for lipid-soluble molecules including unsaturated fatty acids and phytosterols. Ethanol and aqueous ethanol solutions have proven to be highly effective in extracting proteins and co-extracting total phenolic compounds from *T. molitor* and other insect-derived materials. As a result, the extracts that are produced show improved DPPH radical scavenging activity [68]. Despite the technological maturity of conventional solvent extraction, the use of traditional organic solvents may lead to solvent residue risks, while thermal treatment during the extraction process can easily induce the degradation of heat-sensitive bioactive compounds. To address these limitations, green extraction technologies have rapidly developed in recent years, particularly supercritical CO_2_ extraction and deep eutectic solvent-based extraction. Supercritical fluid extraction (SFE) utilizes supercritical CO_2_ as the extraction medium to selectively isolate lipid-soluble bioactive compounds from insects under low-temperature conditions. This approach effectively minimizes the thermal degradation of target bioactive substances [69]. In addition, emerging green solvents, such as deep eutectic solvents (DESs) and ionic liquids, have provided new strategies for the environmentally friendly and efficient extraction of anti-aging bioactive compounds from insect resources [70]. Overall, for water-soluble anti-aging ingredients for mass-market functional foods, conventional aqueous ethanol extraction delivers the best cost–benefit performance; for high-value lipid-soluble actives for premium skincare and supplements, supercritical CO_2_ extraction is the optimal choice despite higher costs; and for mid-to-high-end polar active ingredients, DES-based green extraction offers a balanced solution combining efficiency, environmental friendliness, and activity retention.

#### 3.3.3. Bioprocessing

Biological extraction is an eco-friendly and mild preparation technology that employs enzymes and microorganisms as biocatalysts. Extracts obtained via this approach retain superior bioactivities, including antioxidant capacity, angiotensin-converting enzyme inhibitory activity, and anti-skin-aging properties [71,72,73]. This technology is mainly implemented through three complementary approaches: two preparative technologies (enzymatic extraction, microbial fermentation) and one in vitro evaluation method (simulated gastrointestinal digestion). Enzymatic extraction techniques are divided into three primary types: single-enzyme treatment, dual- or multi-enzyme synergistic treatment, and continuous stepwise enzymatic hydrolysis [48,74]. Microbial fermentation relies on microbe-secreted enzymes to degrade insect substrates and transform active components, achieving extraction and bioactivity modification in one step. This technology is particularly suitable for complex upstream raw materials with refractory components (e.g., chitin-rich whole insect larvae, crude insect oils) that are difficult to process via conventional extraction [26]. In vitro simulated gastrointestinal digestion mimics the sequential physiological environments of the human oral cavity, stomach and small intestine to assess the bioactivity of insect-derived components under physiological conditions [75]. Substantial experimental evidence confirms the performance of biological extraction in preparing insect anti-aging active ingredients. For enzymatic extraction, the key operational parameters for single-enzyme treatment cover a hydrolysis temperature range of 50–60 °C, an optimal pH of 8.0 for Alcalase, an enzyme-to-substrate ratio of 0.5–4% (*w*/*w*), and a reaction duration of 1–24 h. Enzymatic hydrolysis of proteins derived from *T. molitor* and *Alphitobius diaperinus* Panzer efficiently generates bioactive peptides, and the resulting hydrolysates exhibit significantly improved antioxidant activity relative to raw proteins [73]. Dual- or multi-enzyme synergistic treatment, typically combining Alcalase and Flavourzyme, significantly improves the degree of hydrolysis. Compared with single-enzyme treatment, the products derived from this synergistic enzymatic reaction show higher total free amino acid content and stronger antioxidant activity, as measured using ABTS and FRAP assays [46]. Although multi-enzyme formulations incur higher enzyme preparation costs, they produce peptides with higher activity and smaller molecular weight, making them more cost-effective for mid-to-high-end dietary supplements targeting precise anti-aging efficacy [76]. In terms of microbial fermentation, proteases secreted by *Bacillus subtilis* and *Bacillus licheniformis* can efficiently degrade the chitinous exoskeleton of *H. illucens* larvae [54]. Additionally, fermentation of *H. illucens* larvae oil using *Lactobacillus gasseri* and other microorganisms can improve skin permeability, reduce the risk of sensitization, and enrich the product with anti-aging bioactive compounds [26], which directly addresses the unique bottleneck of high chitin content in insect raw materials. Digestion products derived from *Gryllodes sigillatus* Walker and *T. molitor* obtained through this simulation system exhibited potent DPPH and ABTS radical scavenging abilities, as well as prominent inhibitory effects on COX-2 and LOX activities, which are closely related to alleviating chronic low-grade inflammation during aging [32]. In summary, for the large-scale production of protein-based anti-aging peptide raw materials for mass-market foods, single-enzyme extraction offers the optimal cost–benefit ratio; for high-activity peptide products and skincare oil ingredients, multi-enzyme synergistic hydrolysis and microbial fermentation are more suitable despite higher costs; and in vitro simulated gastrointestinal digestion is an indispensable supporting tool for the early development of all oral anti-aging products.

## 4. Potential Mechanisms of Action for Insect-Derived Anti-Aging Agents

**Figure 4:**
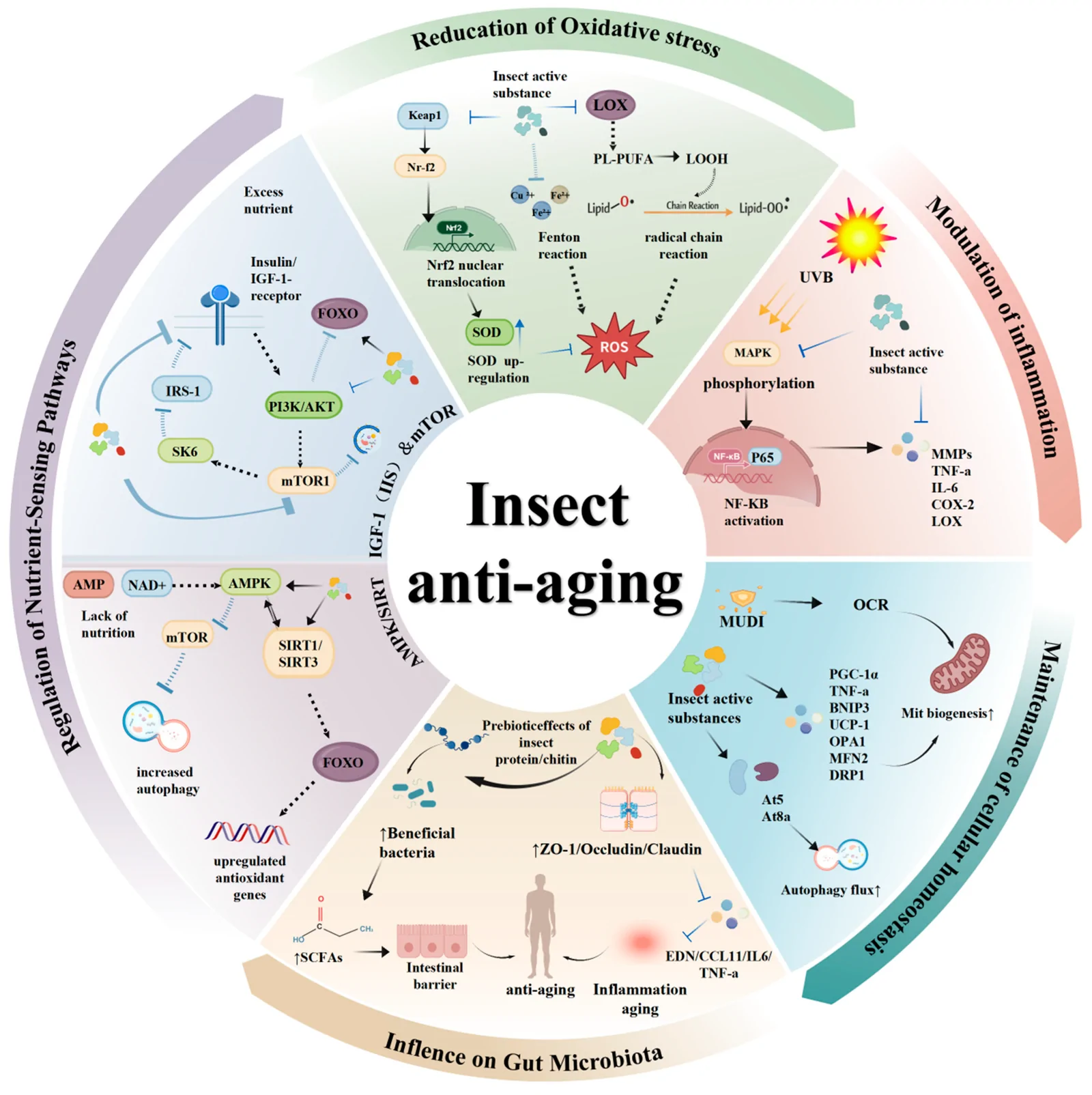
Integrated multi-target and multi-pathway mechanisms of insect-derived bioactive components against aging. Solid arrows indicate experimentally validated pathways supported by in vitro or in vivo evidence, while dashed arrows represent hypothetical, inferred regulatory connections that require further experimental verification.

Insects and their bioactive derivatives can synergistically influence the progression of aging by targeting multiple molecular pathways and signaling mechanisms. The primary mechanisms include regulating nutrient-sensing pathways, reducing oxidative stress, inhibiting inflammatory responses, maintaining cellular homeostasis, and altering the composition of gut microbiota (Figure 4).

### 4.1. Effects on Nutritional Sensing

The nutrient-sensing network functions as a central hub governing cellular metabolism and aging, whose core signaling pathways mainly include the AMPK/SIRT axis, insulin/IGF-1 (IIS) pathway, and mTOR pathway. AMPK acts as a pivotal cellular energy sensor, whereas SIRT1 modulates metabolic processes and stress responses [77]. Collectively, they synergistically maintain cellular energy metabolism and ameliorate cognitive impairment in models. Insect-derived bioactive compounds exert anti-aging effects by targeting multiple nodes across these core nutrient-sensing pathways. First, insect-derived components regulate energy homeostasis and cerebral function via the AMPK/SIRT axis. Aqueous extracts derived from the combined powder of *Zophobas atratus* Fabricius and *T. molitor* efficiently activate the hippocampal AMPK signaling pathway via increasing the p-AMPK/AMPK ratio [78]. In D-galactose-induced aging mice, extracts from *T. molitor* and *P. brevitarsis seulensis* restore the protein levels of SIRT1 and SIRT3 in the prefrontal cortex and hippocampus of aged mice to physiological ranges [14]. Second, the IIS pathway represents an evolutionarily conserved lifespan-extending mechanism in insects. Royal jelly, for example, suppresses the insulin/IGF-1 signaling (IIS) pathway, thereby activating downstream transcription factors including DAF-16/FOXO [79]. Trophallactic fluid secreted by reproductive individuals of *Reticulitermes labralis* Hsia & Fan et Fan contains bioactive compounds that modulate the IIS pathway to mediate lifespan regulation [80]. Third, insect-derived protein hydrolysates delay senescence by inhibiting the PI3K/Akt/mTOR signaling cascade. QBLE, a proteolytic hydrolysate obtained from *A. mellifera* larval royal jelly, prolongs the lifespan of *D. melanogaster* by restraining the PI3K/Akt/mTOR cascade and downregulating the expression of associated kinases and target genes [28]. Collectively, insect-derived bioactive agents cover all three core branches of the nutrient-sensing network, coordinating energy homeostasis maintenance and lifespan control to exert multi-dimensional anti-aging effects.

### 4.2. Alleviation of Oxidative Stress

Oxidative stress arises from excessive accumulation of reactive oxygen species (ROS) during aging, which overwhelms the endogenous antioxidant defense system and disrupts cellular redox homeostasis. This imbalance ultimately triggers oxidative damage to key biological macromolecules, including DNA, lipids, and proteins [81]. Current evidence for insect-derived bioactive components is distributed across two hierarchical levels: chemical-level antioxidant potential in vitro, and phenotypic improvement of aging-related oxidative damage in vivo [43]. Current evidence shows that insect-derived bioactive components alleviate oxidative stress through two main mechanisms: modulation of endogenous antioxidant enzymes, and direct chemical antioxidant actions [82]. In terms of endogenous antioxidant enzyme modulation, insect-derived bioactive components upregulate antioxidant enzyme expression and activity, with supporting evidence from signaling pathway and in vivo studies. Larval royal jelly protein hydrolysate QBLE activates the Nrf2/Keap1 pathway, which further upregulates downstream SOD and CAT expression and lessens lipid peroxidation products [29]. Moving from cellular and molecular mechanisms to in vivo phenotypes, preliminary in vivo evidence from animal models further supports the protective effect of insect-derived components against age-related oxidative damage. Consistently, extracts of *T. molitor* and *P. brevitarsis seulensis* significantly increase the activities of antioxidant enzymes including SOD in the brain and intestinal tissues of aged mice [14]; the major royal jelly protein can alleviate symptoms of Alzheimer’s disease by upregulating SOD1 expression and reducing ROS, RNS, and malondialdehyde levels [83]. In terms of direct antioxidant actions, insect-derived peptides and extracts function via lipoxygenase inhibition, metal ion chelation, and reducing capacity. The synthetic peptide FDPFPK derived from *S. gregaria* displays direct in vitro lipoxygenase inhibitory activity, with an IC50 of 2.58 µg/mL [32]. Hydrophobic amino acid-enriched peptides, including LSPLYE, AGVL, and VAAV, were isolated from hydrolysates of *Gryllus bimaculatus* De Geer. These peptides have hydrophobicity values ranging from 50% to 100%, which is strongly correlated with metal chelating capacity [30]. The aqueous extract of *A. domesticus* exhibited iron-reducing capacity with an EC_1_ value of 12.1 ± 0.7 µM FeSO_4_/mg [53]. Collectively, insect-derived bioactive components act on multiple targets of oxidative stress through the above pathways, jointly constructing an antioxidant defense system to counteract age-related oxidative damage. However, most current evidence remains at the level of chemical antioxidant activity and indirect phenotypic improvement; direct causal evidence linking oxidative stress modulation to lifespan extension at the organism level remains limited and requires further validation in standardized aging animal models.

### 4.3. Alleviating Inflammation

Chronic low-grade inflammation constitutes a core hallmark of aging and is tightly linked to multiple age-associated disorders. COX-2, LOX, and MMPs are pivotal enzymes mediating inflammatory cascades, while NF-κB and MAPK signaling pathways function as core regulatory axes to modulate senescence-associated secretory phenotypes and pro-inflammatory factor expression [36]. Insect-derived bioactive compounds exert synergistic antioxidant and anti-inflammatory effects, enabling multi-target intervention against the core pathological mechanisms underlying inflammatory aging [32]. Two interconnected intervention modes underpin their protective effects: direct suppression of pro-inflammatory enzymes and signaling cascades, and interruption of the oxidative stress-inflammation vicious cycle. First, insect bioactive components directly restrain inflammatory responses by inhibiting pro-inflammatory enzymes and blocking upstream inflammatory signaling pathways. Peptide fractions from *T. molitor*, *S. gregaria*, and *Gryllodes sigillatus* Walker, obtained via simulated gastrointestinal digestion, significantly and concurrently inhibit LOX and COX-2 enzymatic activities, thereby forming a synergistic regulatory network linking antioxidant and anti-inflammatory actions [32]. In the UVB-induced skin photoaging model, larval extracts from *Trypoxylus dichotomus* Linnaeus, *P. brevitarsis seulensis*, *T. molitor,* and *G. bimaculatus* effectively suppress the expression of MMP-1 and MMP-9, thereby protecting dermal collagen from photoaging damage [82]. *G. bimaculatus* extract markedly alleviates alcohol-induced intestinal and hepatic injury by suppressing ROS-mediated oxidative stress and blocking the LPS/TLR4/MAPK signaling cascade [84]. OPA, a low-molecular-weight oligosaccharide purified from the ethanol extraction residues of *P. americana*, markedly inhibits the activation of the TLR4/MAPK/NF-κB signaling cascade, downregulates TLR4 protein expression, and suppresses the phosphorylation levels of p65, p38, JNK, and ERK1/2 [85]. Second, since oxidative stress is a key upstream trigger of NF-κB-mediated inflammatory activation, insect-derived bioactive components block this cascade at the source by scavenging excess free radicals and elevating endogenous antioxidant enzyme activities, thereby disrupting the oxidative stress–inflammation vicious cycle [86]. Collectively, insect bioactive compounds target inflammatory enzymes, core inflammatory signaling axes, and upstream oxidative triggers at multiple nodes, forming a multi-targeted intervention system to alleviate chronic low-grade inflammation and retard inflammatory aging.

### 4.4. Maintenance of Cellular Homeostasis and Repair Capacity

Maintaining intracellular homeostasis and efficient damage repair systems is vital for preserving organelle integrity, resisting cellular stress, and delaying aging [87]. During aging, progressive disruption of cellular homeostasis is driven primarily by two interconnected processes: mitochondrial dysfunction and declining autophagic capacity, both of which accelerate cellular senescence. Insect-derived bioactive compounds exert anti-aging effects through various regulatory mechanisms, including enhancing mitochondrial function and sustaining autophagic homeostasis. Mitochondrial dysfunction is mainly characterized by accumulated mtDNA mutations, disrupted protein balance, impaired mitochondrial turnover, and abnormal mitochondrial dynamics. Insect-derived bioactive components mitigate these impairments to maintain mitochondrial integrity and function. For example, bioactive components from beeswax by-products mitigate mitochondrial oxidative damage in human dermal fibroblasts [52]. Major royal jelly proteins (MRJPs) synergistically improve mitochondrial function through multiple mechanisms, boosting mitochondrial biogenesis, maintaining the homeostasis of mitochondrial dynamics and mitophagy, preserving mitochondrial membrane potential, and regulating antioxidant defense systems, thereby facilitating the differentiation of C2C12 myotubes [88]. Autophagic capacity gradually declines with age, leading to the accumulation of damaged cellular components and accelerated cellular senescence [89]. Modulation of autophagy serves as a crucial mechanism underlying the anti-aging effects of insect-derived bioactive compounds. Notably, the lifespan-extending effect of queen larval protein hydrolysate QBLE is closely linked to the upregulation of core autophagy-related genes *Atg8a* and *Atg5* [28].

### 4.5. Effects on the Gut Microbiome

The progression of aging is closely linked to the diversity of gut microbiota. Dysbiosis of gut microbes increases intestinal barrier permeability, triggers endocrine dysregulation and immune dysfunction, and ultimately exacerbates age-associated tissue damage [87]. Insect-derived proteins, peptides, polysaccharides, and bioactive extracts can be metabolized by gut microbes, remodeling the intestinal microbial composition by selectively enriching beneficial bacteria and suppressing the growth of pathogenic strains. First, insect-derived bioactive substances counteract age-related microbial dysbiosis by modulating community composition. *T. molitor* protein improves gut health in D-galactose-induced aging mice by significantly increasing the abundance of the CHA01 strain and supporting the restoration of gut microbial diversity [25]. Notably, experimental evidence from *D. melanogaster* confirms a microbiota-dependent anti-aging effect: flies lacking 14 antimicrobial peptide genes (*ΔAMP14*) exhibit dysbiosis alongside shortened lifespan. Nevertheless, this lifespan reduction is substantially reversed under germ-free conditions, confirming that insect-derived antimicrobial peptides exert indirect anti-aging effects by maintaining gut microbial homeostasis and preventing age-related dysbiosis [49]. Second, insect bioactive compounds attenuate age-associated intestinal damage by reinforcing intestinal barrier integrity and inhibiting inflammatory responses. Extracts from *P. brevitarsis* and *T. molitor* alleviate intestinal inflammation in aged mice by upregulating the tight junction proteins ZO-1 and Claudin-5, downregulating MMP-2, and inhibiting the pro-inflammatory mediators EDN and CCL11 [14]. Consistently, *P. americana* aqueous extracts mitigate inflammatory responses through the suppression of the TLR4/MyD88/NF-κB cascade, upregulate Occludin-1 and ZO-1 to restore intestinal barrier function, and reshape gut microbiota structure, thereby restraining pathogenic bacteria and promoting the growth of beneficial microflora [90]. Collectively, insect-derived agents target multiple nodes of the gut microbiota–intestinal barrier axis, ameliorating age-related intestinal homeostasis disruption at the microbial community level and the epithelial cellular level to delay the aging process.

## 5. Potential Applications of Insects and Their Bioactive Compounds in Anti-Aging

As a novel and sustainable source of natural functional ingredients, insects contain abundant bioactive substances such as proteins, bioactive peptides, chitin, and phenolic compounds. These substances exert anti-aging effects through multiple mechanisms and demonstrate significant potential for use in both oral and topical formulations.

### 5.1. Oral Anti-Aging Products

#### 5.1.1. Functional Foods

The development of insect-derived functional foods has expanded from traditional beverages to the fields of modern nutritional fortification and precision intervention. Among ready-to-eat products, insect tea is a traditional beverage in the ethnic minority regions of southwest China. Rich in tea polyphenols, it improves multi-organ indices and reduces inflammatory cytokine levels in a D-galactose-induced mouse model of aging, making it one of the few traditional functional foods that already exists in a mature, ready-to-consume form [91]. *T. molitor* protein has been processed into ready-to-eat products such as protein bars and vegetable soups, demonstrating efficacy in ameliorating physiological senescence indicators and improving gut health in aged mouse models [25] (Supplementary Figure S2), thereby providing a sustainable protein supplementation option for elderly individuals with sarcopenia. In the development of medical foods, extracts from *Oecophylla smaragdina* Fabricius and insect-derived ingredients such as *B. mori* and *A. mellifera* are being developed as adjunctive interventions for age-related chronic diseases such as hypertension [33], Parkinson’s disease [92], and Alzheimer’s disease [93,94,95]; most are currently in the formulation development and animal efficacy validation stages.

#### 5.1.2. Dietary Supplements

Dietary supplements represent a key area of development for the concentration and standardization of bioactive compounds derived from insects, and a number of high-potential candidate ingredients have already emerged. Royal jelly is the most commercially mature product in this category; its conventional dosage forms, including capsules, tablets and oral solutions, are widely available on the market; development of QBLE, a hydrolyzed protein derived from queen bee larvae extract, is also progressing steadily. In addition, multiple bioactive components are in the transition phase from efficacy validation to commercial product development [28]. For example, extracts from *T. molitor* and *P. brevitarsis seulensis* are being developed as neuroprotective supplements [14]; selenium-enriched *T. molitor* peptides, due to their enhanced immunomodulatory activity, show promise as high-end immune support dietary ingredients [24]; IDA (trans-3-indoleacrylic acid), a natural compound identified in the trophallactic fluid exchanged among different castes of the *R. labralis*, has shown preliminary potential as a candidate for precision longevity supplements, owing to its ability to target conserved aging-regulatory pathways such as IIS and RAS [80] (Supplementary Figure S2). Although the bioactivity of these raw materials has been validated in experimental models, their industrialization remains constrained by formulation development and long-term safety assessments; they are currently in the transition phase from raw materials to standardized end products.

### 5.2. Topical Anti-Aging Products

#### 5.2.1. Protective Products

Insect-derived active ingredients have demonstrated significant protective potential against UV-induced skin damage. Silkworm pupa peptides alleviate the process of photoaging by reducing oxidative stress and regulating immune function [13] (Supplementary Figure S2), while fermented *H. illucens* larval oil is better suited for the development of age-friendly skincare products [26]. However, these ingredients are currently in the raw material validation stage, with no commercial products yet available on the market, and human clinical evidence for their photoprotective anti-aging efficacy is still insufficient.

#### 5.2.2. Repair Products

Collagen and elastin degradation is the core driver of skin wrinkles and laxity. Multiple studies have confirmed that insect-derived active ingredients can effectively inhibit key hydrolytic enzymes in this process. At the raw material level, *A. domesticus* water extracts possess dual functions: inhibiting MMP-1-mediated collagen degradation and promoting TGF-β1-mediated collagen synthesis, without causing skin irritation [96]; *A. mellifera* larva protein and its hydrolysate also demonstrate excellent anti-collagenase and anti-hyaluronidase activity [71] (Supplementary Figure S2). At the product level, bee venom serums have shown clear efficacy in reducing wrinkle area, number, and depth in clinical studies involving healthy women [97]; cosmetic formulations containing *T. molitor* oil have been incorporated into anti-wrinkle patents [7]. Overall, the collagenase/elastase inhibitory activity of most insect-derived skincare ingredients has only been verified in vitro, and large-scale clinical evidence for in vivo anti-wrinkle efficacy remains limited, with only a few ingredients progressing to clinical evaluation.

## 6. Challenges and Future Prospects

As the most diverse and renewable biological taxon on Earth, insects offer a promising alternative to conventional anti-aging resources [14] and show significant potential for use in functional foods, nutritional supplements, and cosmetics. Bibliometric analyses reveal that research in this field has grown rapidly since 2013, with the annual number of publications reaching 81 in 2022. This trend indicates that insect-derived anti-aging research has become a burgeoning interdisciplinary frontier connecting food science and geroscience. Recently, increasing attention has focused on key bioactive compounds isolated from insects, such as proteins and peptides [13], lipids [22], polyphenols [91], polysaccharides [45], and trace elements [24]. Researchers have systematically studied their extraction and purification methods, structural characteristics, and anti-aging activities. Additionally, insects offer several practical advantages, including broad distribution, low breeding costs, and rapid reproductive rates. These features, combined with their diverse biological activities, allow insects to deliver multiple anti-aging effects, including antioxidant defense, anti-inflammatory action, immune regulation, promotion of collagen synthesis, and modulation of the gut microbiota. As a result, insect-derived bioactive compounds present strong potential for large-scale development and industrial application. Despite the substantial progress achieved in elucidating the anti-aging properties of insects, several challenges continue to hinder further research and industrial translation.

### 6.1. Germplasm Mining Bottlenecks and Screening Strategies

Significant limitations remain in the exploration of insect resources for anti-aging applications. To date, only 32 insect species have been characterized with respect to their anti-aging mechanisms and related bioactive constituents, whereas numerous other species with potential anti-aging properties have yet to undergo systematic bioactivity screening and component identification. Notably, this count of 32 species is derived exclusively from published peer-reviewed studies and does not encompass folk or regional ethnomedicinal uses of insects for anti-aging and health-preserving purposes. This slow progress stems from three insect-specific root causes rather than general screening workload. First, the existing large-scale insect breeding system is optimized for biomass yield and total protein content for feed and food applications [74], while targeted breeding and supporting research toward the enrichment of anti-aging bioactive components remains largely underexplored. Second, as metamorphic organisms, insect metabolite profiles change dramatically across developmental stages [98], yet conventional rearing and screening practices lack standardized harvesting windows defined by active component content. Third, a considerable proportion of bioactive components are derived from insect gut symbionts rather than the insect itself [99], and uncontrolled symbiotic flora variation directly translates into product compositional differences. As a result, the developmental potential of insect resources remains largely untapped (Supplementary Table S1).

To bridge this gap and fully unlock the anti-aging potential of insect germplasm resources, a full-chain development path covering high-throughput germplasm screening, targeted breeding optimization and industrial scale-up promotion should be constructed. A targeted, insect-adapted technology-integrated pipeline encompassing multi-omics profiling [100], network pharmacology [101], artificial intelligence [102], and high-throughput screening in model organisms [103] is thus required for efficient, large-scale mining of bioactive insect candidates. At the experimental profiling level, dynamic multi-omics analysis covering key developmental stages and representative rearing conditions, integrating metabolomic and proteomic analyses, enables systematic compositional mapping of diverse and medicinal insect species, facilitating the precise identification of core bioactive constituents including flavonoids, polyphenols, peptides, and polysaccharides. When coupled with network pharmacology approaches that integrate both host endogenous components and symbiotic microbial metabolites, these identified molecules can be mapped to canonical anti-aging signaling hubs, including FOXO, SIRT1, Nrf2, mTOR, and AMPK [104], enabling the prioritization of elite insect species with broad target coverage and synergistic component interactions. For targeted breeding optimization, a breeding–processing–bioactivity (BPB) coupling framework should be established, incorporating dietary nutrient ratios [24], developmental harvesting windows [105], environmental stress induction [106], and symbiotic flora regulation [99] into the quality control system. This enables directional enrichment of anti-aging components through precise rearing parameter tuning. For industrial scale-up, species-specific standardized breeding protocols for medicinal insects, full-chain quality traceability systems aligned with food and pharmaceutical regulations [107], and low-cost automated breeding technologies [108] are essential prerequisites for expanding cultivation and commercial translation. Notably, several insect-derived anti-aging products have already achieved commercial maturity—including royal jelly, insect tea, and QBLE—providing a practical foundation for the broader exploitation of insect anti-aging resources.

### 6.2. Quality Stability Bottlenecks and Optimization Strategies

Ensuring the quality and stability of insect-derived products remains a major challenge. At the industrial production level, the composition and concentration of insect-derived bioactive compounds are affected by multiple variables from breeding to processing, further amplifying the batch-to-batch variability observed in basic research. As a result, substantial batch-to-batch variability frequently occurs, creating a significant obstacle to the standardized production and rigorous quality control of insect-based pharmaceuticals and functional health products [7]. For example, the qualitative and quantitative profiles of polyphenols in *A. domesticus* and *T. molitor* are highly dependent on dietary conditions. Even individuals belonging to the same insect species may exhibit marked differences in the composition of polyphenols and phenolic derivatives [43]. In addition to compositional instability, the biological stability of insect-derived bioactive compounds also remains problematic. Key active substances, such as peptides and polysaccharides, are highly susceptible to gastrointestinal degradation and oxidative inactivation on the skin surface, which may significantly reduce their bioavailability and therapeutic efficacy. Therefore, advanced delivery technologies, including nanoencapsulation [9] and liposomal carrier systems, are considered essential for improving their structural stability and biological activity [109]. However, most existing studies directly adopt mature delivery technologies from the food and pharmaceutical industries without targeted adaptation to the unique structural features of insect-derived molecules, thereby restricting their practical industrial application and large-scale commercialization. Accordingly, future research must adopt a multi-pronged, system-level approach.

First, a processing–property–activity relationship framework [110] analogous to the well-established “process-structure-function” matrix for other biopolymers should be established for insect-derived bioactive compounds, incorporating upstream rearing parameters (dietary formulation, developmental harvesting window, symbiotic flora regulation) into the analytical system. This framework will enable systematic deciphering of the molecular drivers underlying compositional variability, shifting quality control from passive end-point detection to proactive upstream process design. Second, elucidating the precise molecular degradation pathways (e.g., enzymatic cleavage sites in the gastrointestinal tract, pH-dependent hydrolysis patterns) of insect-derived peptides and polysaccharides is a prerequisite for rational stabilization strategies, such as site-specific amino acid substitution or targeted chemical modification [111]. While these approaches have been successfully implemented for synthetic and food-derived bioactive peptides, their application to insect-derived anti-aging peptides remains largely unexplored and warrants systematic investigation. Third, the delivery efficiency of bioactive compounds needs further optimization. Targeted modification of nanocarriers based on the molecular weight and polarity characteristics of insect active substances can be combined with advanced self-assembly technologies to construct innovative delivery and controlled-release systems. Such strategies effectively improve the bioavailability and anti-aging efficacy of insect bioactive compounds, enabling personalized nutritional supplementation [112].

### 6.3. Mechanistic Research Bottlenecks and Translational Innovation Strategies

Current mechanistic studies on insect-derived anti-aging compounds are still underdeveloped, a lag largely driven by the mismatch between the unique attributes of insect bioactive systems and conventional single-target research paradigms, rather than simply insufficient research investment. Most existing research primarily focuses on observing aging-related phenotypic changes [14], while the underlying molecular targets remain unclear and lack experimental validation. This is largely attributed to the multi-component synergistic action mode of insect extracts: traditional activity-guided single-component purification often disrupts the synergistic effect and leads to decreased bioactivity, which greatly hinders the identification of core effector molecules and their direct targets. Furthermore, the functional verification of key regulatory genes remains inefficient, as advanced genetic techniques such as gene knockouts and overexpression analyses have not been widely applied. As a result, most investigations into the anti-aging mechanisms of insect-derived bioactive compounds are largely limited to phenotypic characterization, impeding the development of a comprehensive theoretical framework for precise regulatory intervention and mechanistic understanding.

To address these limitations, future research should establish a rigorous stepwise pipeline tailored to insect-derived bioactive systems to bridge the gap between phenotypic observation and translational application, rather than relying on fragmented trial-and-error studies. Activity-guided fractionation combined with high-resolution mass spectrometry [101] and nuclear magnetic resonance spectroscopy [10] is required to identify bioactive molecule groups with retained synergistic effects, followed by advanced chemical biology approaches to elucidate their direct protein targets, elevating mechanistic understanding from pathway-level correlation to precise binding interactions. A two-tier verification strategy should be adopted: CRISPR/Cas9-mediated gene knockout is first performed in representative model insects for high-throughput causal validation of identified targets, providing definitive causal evidence linking target modulation to anti-aging efficacy. Building on validated mechanistic findings, researchers can integrate AI-assisted in silico screening, CRISPR gene editing [113], and sericin/chitosan hydrogel encapsulation [114] to construct insect-derived bioactive factor delivery systems based on sericin/chitosan hydrogel encapsulation, enabling sustained and tunable release of anti-aging effectors in vivo [9]. A tiered in vivo assessment framework covering accelerated aging models and naturally senescent/progeroid models should be adopted, with multi-dimensional phenotyping and head-to-head comparison to provide more reliable preclinical evidence to support [14,25]. This targeted sequential pipeline can systematically resolve the current mechanistic ambiguities in insect anti-aging research, laying a solid scientific foundation for the rational development and clinical translation of high-value insect-derived anti-aging products.

### 6.4. Extraction Technology Bottlenecks and Industrial Scale-Up Strategies

One of the major challenges facing current extraction technologies is the substantial gap between laboratory-scale research and industrial-scale production, which is particularly pronounced for insect-derived bioactive ingredients due to the unique structural and compositional properties of insect raw materials. Conventional extraction methods, such as water extraction and ethanol precipitation, often exhibit low extraction efficiency and high impurity levels, limiting their suitability for large-scale manufacturing. To overcome these limitations, several emerging green extraction technologies, including microwave-assisted extraction, ultrasonic-assisted extraction, and supercritical CO_2_ extraction, have been introduced into this field [43,58,69]. However, these advanced approaches still face significant challenges, particularly inadequate process optimization and insufficient standardization [63]. Furthermore, most existing extraction and purification protocols apply only to small-scale laboratory preparation. This compositional heterogeneity, originating from upstream breeding and developmental differences, directly translates to unstable end-product efficacy and creates a core barrier to standardized formulation and mass production. As a result, standardized manufacturing procedures capable of ensuring stable product quality, high experimental reproducibility, and effective quality control have not yet been fully established [102]. To address these systemic limitations and bridge the lab-to-industry translation gap, isolated improvements to individual equipment parameters are far from sufficient; instead, a three-dimensional upgrade covering process concept, optimization methodology and production mode is required.

First, the single-target extraction paradigm should be replaced by a “biorefinery” concept. Given that insect biomass naturally coexists with multiple high-value components, a sequential fractionation workflow that recovers lipids, proteins, polysaccharides and chitin in turn from the same biomass can generate multiple high-value product streams, which has proven economically viable in microalgae processing and agricultural [115] residue valorization and represents a key R&D direction for high-biomass insect species such as *T. molitor*. Second, empirical one-factor-at-a-time experimentation should be replaced by a data-driven optimization framework. Response surface methodology [116], artificial neural networks [117] and kinetic modeling should be systematically applied to quantify multi-variable interactions and critical engineering parameters, while raw material attribute parameters (such as instar stage and crude composition) should be incorporated into the model to improve process adaptability to the heterogeneity of insect raw materials, filling the current gap in process scale-up data for insect bioactive extraction.

### 6.5. Safety Control Bottlenecks and Precision Mitigation Strategies

Product development and safety management remain relatively underdeveloped; these industrialization challenges are particularly pronounced for insect-derived anti-aging ingredients, as they are deeply intertwined with the distinct biological properties of insect raw materials and the current maturity of efficacy evidence. Current research has primarily focused on crude extracts, hydrolysates, and purified peptides, whereas commercially applicable products, such as topical creams, essences, serums, oral tablets, capsules, and functional beverages, are still insufficiently developed [118]. The inherent batch-to-batch variability of active components, driven by insect developmental stages and rearing conditions, is a core underlying cause limiting formulation standardization and mass production. Consequently, the industrial translation and market application of insect-derived anti-aging compounds remain limited. At the same time, several potential safety concerns have not yet been fully addressed. As an emerging category of bioactive raw materials for anti-aging use, insect-derived components still lack a dedicated safety evaluation system and full-chain control standards adapted to their production characteristics [108]. These risks are not isolated: endogenous allergens are inherent structural proteins of insects, while heavy metals and microbial contaminants are mainly bioaccumulated through rearing substrates, together forming a full-chain risk system. For example, insect proteins such as tropomyosin and arginine kinase share more than 60% sequence homology with allergens derived from crustaceans and dust mites, thereby increasing the risk of cross-allergic reactions [119]. In addition, heavy metal contamination, pesticide residues, and microbial pollution require strict monitoring through standardized breeding practices and rigorous processing procedures [120].

Allergenicity and contaminant risks represent core safety barriers for the industrialization of insect-derived products, necessitating a full-chain tiered precision control framework rather than sole reliance on terminal batch testing. For allergenicity control, targeting insect-characteristic allergens (tropomyosin, arginine kinase), a three-level gradient control strategy is implemented across source-processing-formulation. In silico deimmunization tools and epitope mapping guide targeted site-directed mutagenesis or enzymatic clipping of major allergens such as tropomyosin and arginine kinase at the source level, reducing immunoreactivity while requiring validation of preserved native protein bioactivity [121]. During downstream processing, aptamer-based affinity chromatography and immobilized metal affinity chromatography enable highly selective removal of trace residual allergens from complex hydrolysates, outperforming conventional solubility-based fractionation. In final product formulations, epitope masking strategies including polysaccharide covalent conjugation and inert polymer encapsulation, adapted from pharmaceutical protein immunogenicity reduction approaches, physically shield immunoreactive sites to provide an additional safety layer for both oral and topical applications [122]. For chemical and microbial safety, in response to the feed-driven bioaccumulation feature of insect contaminants, the control gateway is shifted forward to the rearing stage to block contaminant bioaccumulation at the source. Integrated sensor networks comprising surface-enhanced Raman spectroscopy [123], laser-induced breakdown spectroscopy [122], and electronic nose [124] systems enable real-time, non-destructive contamination monitoring at key production nodes, with strong translational foundations from proven applications in environmental monitoring and advanced manufacturing. At the feed source, low-dose functional additives such as modified zeolites and engineered biochar selectively sequester heavy metals and organic contaminants in substrates, proactively blocking their bioaccumulation in insect tissues before entry into the production chain [125]. Furthermore, machine learning models integrated with full-chain process data covering rearing, extraction, and formulation enable probabilistic forecasting of microbial proliferation risks [126], triggering pre-emptive interventions and upgrading safety management from passive end-point testing to active predictive control.

## 7. Conclusions

With the accelerating aging of the global population, developing safe, efficacious, and sustainable anti-aging interventions has become a critical research priority in life sciences and public health. Insects, the most diverse and renewable biological resource on the planet, have expanded their application beyond traditional food and feed use, emerging as promising bioresources for anti-aging research. Bioactive components derived from insects—including proteins, peptides, polyphenols, and polysaccharides—exert well-documented anti-aging activities by modulating nutrient-sensing pathways, reducing oxidative stress, alleviating inflammation-induced senescence, and maintaining cellular homeostasis. These multifunctional compounds exert synergistic multi-target regulatory effects that align well with the complex nature of aging, making them highly promising for both oral and topical anti-aging applications. However, significant challenges remain in translating insect-derived bioactive ingredients into clinical translation and industrial scale-up of insect-derived bioactive ingredients. Advancing the field requires three core pillars: in-depth elucidation of molecular mechanisms, standardized preparation and quality control systems for active ingredients, and well-designed clinical trials with robust endpoints. Continued advancements in insect-based anti-aging research will not only offer safe, natural approaches to promote healthy aging and extend healthspan but also pave new avenues for the development of sustainable, low-carbon health industries.

## Figures and Tables

**Table 1:** Functional substances and action mechanisms in common anti-aging insect species.

Bioactive Components	Specific Components	Insects	Characteristic Structure/Sequence	Associated Anti-Aging Phenotype/Pathway	Ref.
Protein/ Peptide	Antioxidant peptide	*Schistocerca gregaria* Forsskål	FDPFPK	ABTS radical scavenging, DPPH radical scavenging, LOX **↓** COX-2 **↓**	[32]
		*Schistocerca gregaria* Forsskål	AIGVGAIER	ABTS radical scavenging, DPPH radical scavenging, LOX **↓** COX-2 **↓**	[32]
		*Oecophylla smaragdina* Fabricius	CTKKHKPNC	ABTS radical scavenging DPPH radical scavenging	[33]
		*Hermetia illucens* Linnaeus	GYGFGGGAGCLSMDTAGAHLNR, VVPSANRAMVGIVAGGGRIDKPILK, AGLQFPVGR, GFKDQIQDVFK, etc.	ABTS/DPPH radical scavenging	[34,35]
		*Bombyx mori* Linnaeus	SPPs	ROS **↓**, MDA **↓**, SOD **↑**, GSH-Px **↑**, CAT **↑**, IL-6 **↓**, IL-10 **↑**, Hyp **↑**, MMP-1 **↓**,	[13]
		*Gryllus bimaculatus* De Geer	GA-2	ABTS radical scavenging, metal chelating ability	[30]
	Protein Hydrolysate	*Hermetia illucens* Linnaeus	BPHs	ROS **↓**, Nrf2 nuclear translocation **↑**	[31]
	Proteins	*Bombyx mori* Linnaeus	SF	ROS **↓**, MDA **↓**, SOD **↑**, GSH-Px **↑**, AP-1 **↓**, MPKA **↓**, MMP **↓**	[36]
		*Ulomoides dermestoides* Chevrolat	UGP2A	IL-6 **↓**, TNF-α **↓**, iNOS **↓**, COX **↓**, Nuclear translocation of NF-κB **↓**	[37]
		*Apis mellifera* Linnaeus	MRJPs	IIS **↓**, TOR **↓**, EGF **↑**	[38]
Lipids	MUFA	*Apis mellifera* Linnaeus	10-had	IIS **↓**, TOR **↓**, EGF **↑**, HDAC **↓**	[39]
	PUFAs/MUFAs/SFA	*Hermetia illucens* Linnaeus	HIO/FHIO	Collagen synthesis **↑**, tyrosinase activity **↓**	[26]
Polyphenols	Phenolic Derivative	*Luprops tristis* Fabricius	2,5-dimethylhydroquinone; 1,3-dihydroxy-2-methylbenzene	DPPH, ABTS free radical scavenging	[40,41]
	Phenolic Acids	*Acheta domesticus* Linnaeus	4-Hydroxybenzoic acid, p-Coumaric acid, ferulic acid, and Syringic acid	DPPH IC_50_ 0.28–0.35 mg/mL); ABTS IC_50_ (0.46–0.74 mg/mL)	[42,43]
Polysaccharides	Cordyceps Cicadae Polysaccharides	*Paecilomyces cicadae* (Miq.) Samson	CP70	Drosophila lifespan **↑**, CAT/SOD1/MTH **↑**, MDA **↓**	[44]
	Glycosaminoglycan	*Catharsius molossus Linnaeus*	GAG	Antioxidant pathway, lipid metabolism regulation, matrix remodeling	[23]
	Chitosan	*Clanis bilineata* Tsingtauica	CBLSWSC	SOD **↑**, GSH-Px **↑**, MDA **↓**	[45]
Trace elements	Se	*Tenebrio molitor* Linnaeus	Se	GPx activity **↑,** antioxidant defense **↑**, immunomodulation **↑**	[24]

## Data Availability

This article is a review and does not include new experimental data. All data cited and discussed in the manuscript are derived from previously published studies, which are appropriately referenced throughout the text. No new datasets were generated or analyzed during the preparation of this work.

## Supplementary Materials

The following supporting information can be downloaded at https://www.mdpi.com/article/10.3390/insects17080847/s1. Figure S1: Bibliometric visualization map: (A) Sankey diagram: Authors, Keywords, and Affiliations; (B) Keyword timeline map; Figure S2: Multi-scenario Application of Insect-derived Active Ingredients in Anti-aging Intervention; Table S1: A Systematic Overview of Anti-Aging Insects: Active Ingredients, Extraction Methods, and Experimental Models [127,128,129,130,131,132,133,134,135,136,137,138,139,140,141,142,143,144,145,146,147,148,149,150,151,152,153,154,155].

## Abbreviations

The following abbreviations are used in this manuscript:

CAT	Catalase
COX-2	Cyclooxygenase-2
GSH-Px	Glutathione peroxidase
HDAC	Histone deacetylase
IIS	Insulin/insulin-like growth factor signaling pathway
LOX	Lipoxygenase
MDA	Malondialdehyde
MMP-1	Matrix metalloproteinase-1
Nrf2	Nuclear factor erythroid 2-related factor 2
ROS	Reactive oxygen species
SOD	Superoxide dismutase
TGF-β1	Transforming growth factor-β1
TOR	Target of rapamycin
**↑**	Upregulation/enhancement
**↓**	Inhibition/reduction
