# Supplementary material for: Insect-Derived Anti-Aging Bioresources: Current Status, Bottlenecks, and Future Directions
Source: Insects. 2026 Aug 14;17(8):847. doi: 10.3390/insects17080847 (PMC13513282; doi:10.3390/insects17080847)
Supplement: Supplementary file 1 [file insects-17-00847-s001.zip › insects-4428055-supplementary.pdf]

Supplementary Material

Supplementary Figure S1: Bibliometric visualization map:(A) Sankey diagram:Authors, Keywords and Affiliations;(B) Keyword timeline map

A

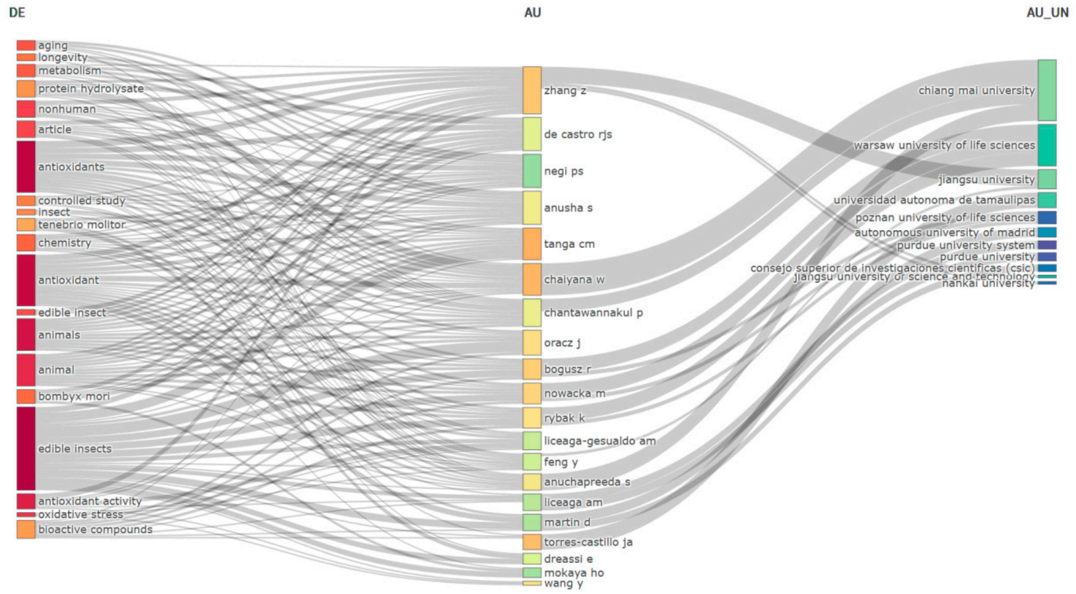

B

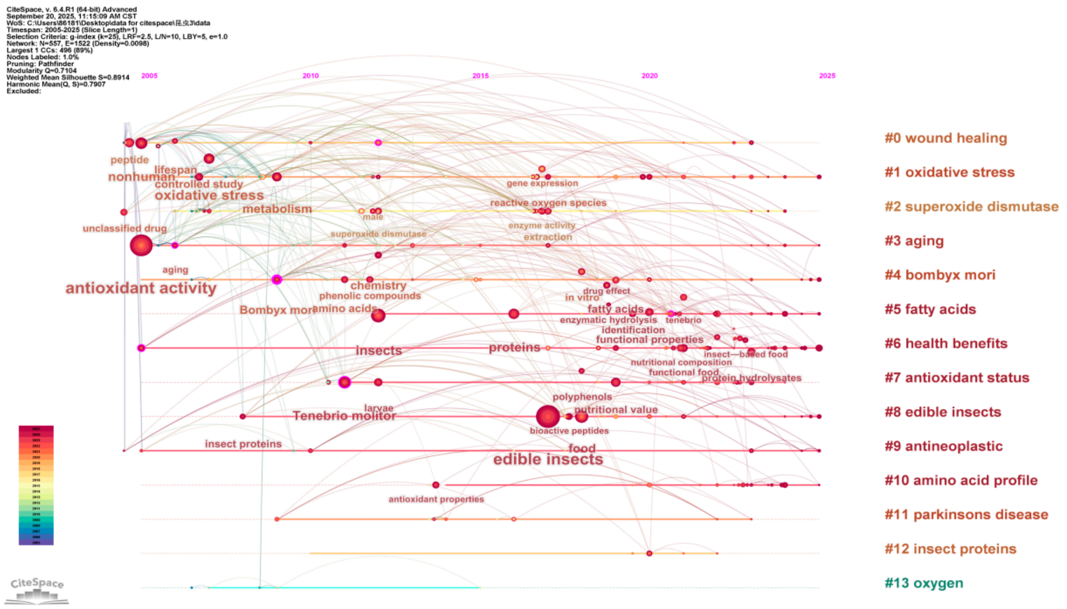

Supplementary Figure S2: Multi-scenario Application of Insect-derived Active Ingredients in Anti-aging Intervention

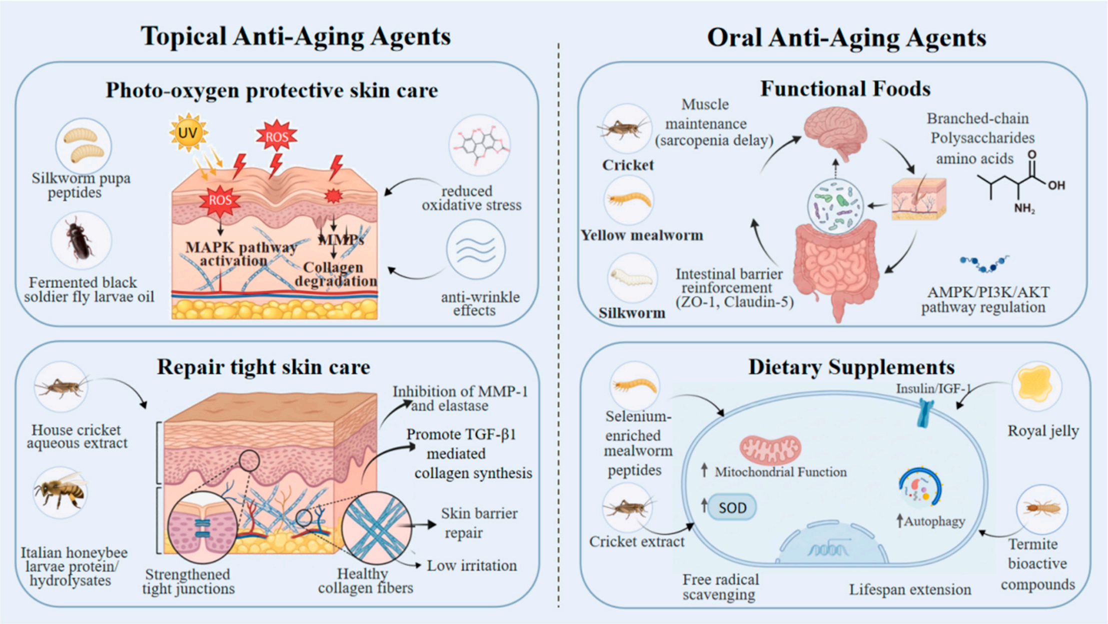

Topical Anti-Aging Agents
Photo-oxygen protective skin care
Silkworm pupa peptides
Fermented black soldier fly larvae oil
UV
ROS
MAPK pathway activation
MMPs
Collagen degradation
reduced oxidative stress
anti-wrinkle effects
Repair tight skin care
House cricket aqueous extract
Italian honeybee larvae protein/ hydrolysates
Inhibition of MMP-1 and elastase
Promote TGF-β1 mediated collagen synthesis
Skin barrier repair
Low irritation
Strengthened tight junctions
Healthy collagen fibers
Oral Anti-Aging Agents
Functional Foods
Muscle maintenance (sarcopenia delay)
Cricket
Yellow mealworm
Silkworm
Branched-chain Polysaccharides amino acids
Intestinal barrier reinforcement (ZO-1, Claudin-5)
AMPK/PI3K/AKT pathway regulation
Dietary Supplements
Selenium-enriched mealworm peptides
Cricket extract
Insulin/IGF-1
Royal jelly
Mitochondrial Function
SOD
Autophagy
Termite bioactive compounds
Free radical scavenging
Lifespan extension

Supplementary Table S1: A Systematic Overview of Anti-Aging Insects: Active Ingredients, Extraction Methods, and Experimental Models

Catalogue	Species	Medicinal Form	Active Ingredients	Extraction Method	Experimental Model	Ref
Coeoptera	*Tenebrio molitor* Linnaeus	Adult/Larva	Whole Insect Protein	Isoelectric precipitation	Caenorhabditis elegans model	[127]
			Bioactive Peptides, Phenolics	Water extraction	Mice with D-galactose-induced aging model	[25]
			Lipids	Soxhlet extraction method	—	[105]
	Alphitobius diaperinus Panzer	Larva	Protein-polyphenol Conjugates	Alkaline covalent coupling method; Alkaline dissolution-isoelectric precipitation method	*In vitro* antioxidant assay	[128]
			Proteins	Enzymatic hydrolysis	*In vitro* antioxidant assay (ABTS,ORAC)	[73]
	Ulomoides dermestoides Fairmaire	Larva	Polyphenols	Organic solvent extraction method	*In vitro* cell assay (HaCaT cells)	[129]
		Adult	Lipids	Soxhlet extraction	Diabetic model mice	[130]
		—	Glycoprotein (UGP2A)	Water extraction and ethanol precipitation method, combined with DEAE-ff and Sephadex G-100 column chromatography	*In vivo:* Deep vein thrombosis (DVT) mouse model *In vitro*: Human umbilical vein endothelial cells (HUVECs)	[131]
		Larva	Water Extract	Water extraction method	Mouse neurotoxicity model of Parkinson's disease	[92]
	Holotrichia parallela Motschulsky	Adult	Polyphenols	Solvent extraction method	*In vitro* antioxidant activity evaluation model	[22]
	Protaetia brevitarsis seulensis Kolbe	Larva	Flavonoids	Water extraction	Mice with D-galactose-induced aging model	[14]

Catalogue	Species	Medicinal Form	Active Ingredients	Extraction Method	Experimental Model	Ref
	Allomyrina dichotoma Linnaeus	Larva	Polyphenols	Organic solvent extraction method	*In vitro* chemical model	[132]
	Polyphylla adspersa Motschulsky	Larva	Polypeptides	Combined method of enzymatic hydrolysis and separation and purification	Antioxidant enzyme activity determination; intracellular ROS detection	[133]
	Zophobas morio Fabricius	—	Protein Hydrolysates	Enzymatic hydrolysis	*In vitro* chemical model & *in vitro* gastrointestinal digestion model	[134]
	Catharsius molossus	Adult	GaGs	Ethanol extraction	14-month-old male aged SD rat model	[23]
	Blaps rhynchopetera Fairmaire	Adult	Flavonoids,Polyphenols	Water extraction method,Ethanol extraction method	—	[101]
Orthoptera	*Schistocerca gregaria* Forsskål	Adult	Peptides	Water extraction followed by enzymatic hydrolysis	*In vitro* antioxidant activities	[15]
	Acheta domesticus Linnaeus	Adult	Proteins，Polyphenols，Flavonoids	Heat-assisted solvent extraction	Evaluation model of anti-skin aging activity	[96]
		—	Extract	Maceration and Digestion:	*In vitro* antioxidant models, *In vitro* anti-aging models, HET-CAM assay, Human patch test	[53]
	Gryllodes sigillatus Fabricius	Adult	Polypeptides	Enzymatic hydrolysis method	*In vitro* experimental model	[135]
	Gryllus bimaculatus De Geer	Adult	Proteins/ Peptides	Enzymatic hydrolysis method	*In vitro* chemical antioxidant model	[30]
Hymenoptera	*Apis mellifera* Linnaeus	QBL	QBL Protein Hydrolysate	Enzymatic hydrolysis method;Aqueous extractionWater extraction method	*In vitro* biochemical enzyme inhibition assay and hen's egg chorioallantoic	[28, 71]

Catalogue	Species	Medicinal Form	Active Ingredients	Extraction Method	Experimental Model	Ref
					membrane（HET-CAM) irritation test; Drosophila melanogaster lifespan and locomotor activity model	
		RJ	Royal Jelly Total Extract	—	*In vitro* biochemical model	[136]
		Bee Venom	Bioactive Peptides	High Performance Liquid Chromatography	*In vitro* chemical antioxidant assays	[137]
		Beeswax	By-Product MUD1 (polyphenols and flavonoids)	—	*In vitro* experiment of human dermal fibroblasts (HDF)	[52]
		Propolis	Polyphenols, Flavonoids	Natural deep eutectic solvent extraction method	Chronological lifespan model of yeast	[138]
	Polyrhachis vicina Roger	Adult	Polyphenols, Lipids	Ultrasound-microwave assisted ethanol extraction method	*In vitro* antioxidant assay(DPPH, ABTS and hydroxyl radical scavenging assays, ferric ion reducing antioxidant power)	[139]
	Oecophylla smaragdina Fabricius,	Larva and Pupae	Proteins, Polypeptides	antioxidant peptides (CTKKHKPNC) ACE-inhibitory peptides（FFGT and LSRVP)	*In vitro* activity evaluation and determination	[33]
	Bombus Ignitus Smith	Adult	GaGs	—	High-fat diet-induced obese rat model	[140]
Lepidoptera	*Bombyx mori* Linnaeus	Silkworm Pupa	Silkworm Pupa Oil	—	*In vivo* animal model	[51]
		Silk	Sericin	—	*In vitro* fibroblast culture model	[141]
		Cordyceps militaris	Cordycepin	—	*Caenorhabditis elegans* aging model	[142]

Catalogue	Species	Medicinal Form	Active Ingredients	Extraction Method	Experimental Model	Ref
			Polysaccharides	Water extraction and alcohol precipitation method	Mice with D-galactose-in duced aging model	[143]
			Cordyceps militaris mycelial Polysaccharide-ir on Complex	Water extraction -alcohol precipitation-co mplexation method	—	[144]
	Spodoptera littoralis Boisduval	6rd Instar Larvae	Bioactive Peptides	Simulated gastrointestinal digestion method	*In vitro* enzymatic hydrolysis model and *in vitro* activity determination	[145]
	Clanis bilineata Walker	5rd Instar Larvae	Chitosan	Enzymatic hydrolysis method	D-galactose-in duced aging mouse model	[45]
	Galleria mellonella Linnaeus	Larva-pupa TransitionAl stage	Antimicrobial Peptides	Induced extraction and chromatographic purification method and Water extraction -alcohol precipitation method	*In vitro* antibacterial experiment	[146]
Blattodea	*Periplaneta americana* Linnaeus	Adult	Various Fatty Acids	GC-MS	Determination of chemical antioxidant capacity	[147]
			Polypeptides	Enzymatic hydrolysis and ultrafiltration	Antioxidant experiment, anti-inflammat ory experiment, cell proliferation promotion and wound healing experiment	[86]
			Polysaccharides (PAP55-3-1)	Alkaline extraction and Ethanol precipitation and Column chromatography	*In vitro* and *in vivo* experiments	[10]

Catalogue	Species	Medicinal Form	Active Ingredients	Extraction Method	Experimental Model	Ref
	Eupolyphaga sinensis Walker	Dried Female Adults	Polypeptide Extracts	Directed enzymatic hydrolysis technology	*In vitro* antioxidant assay,*in vivo* mousephotoaging model Mouse oxidative stress aging model.	[148]
	Reticulitermes labralis Hsia & Fan et Fan	Ants of different castes perform trophallaxis, the mouth-to-mouth exchange of oral fluid	IDA	Organic solvent extraction method	Metabolomics and *in vitro* functional validation	[80]
	Macrotermes subhylanus Rambur	Adult	Polyphenols	Water extraction -alcohol precipitation method	*In vitro* antioxidant assay	[149]
Hemiptera	*Dactylopius coccus* Costa	—	CA	Methanol extraction method	*In vitro* chemical models: DPPH· radical scavenging, $ABTS^{+}$ radical scavenging; in vitro simulated lipid oxidation model	[150]
	Encosternum delegorguei Spinola	Adult	Flavonoids	Methanol-water extraction method	—	[151]
Diptera	*Musca domestica* Linnaeus	3rd Instar Larvae	Chitosan	Chemical method	*In vitro* experimental model	[152]
			Larva Hemolymph	Anterior end amputation collection method	*In vitro* cell assay,*in vivo* induction experiment	[153]
	Hermetia illucens Linnaeus	Larva	Proteolytic Peptides	Enzymatic hydrolysis method	*In vitro* antioxidant experiment	[29, 31]
	Lucilia	Larva	MAE-P6	Alkaline buffer	Rat deep	[154]

Catalogue	Species	Medicinal Form	Active Ingredients	Extraction Method	Experimental Model	Ref
	sericata Meigen			or salt solution, Ultrafiltration technology	second-degree scald burn model	
	Chrysomya albiceps Wiedemann	Larva	Lipids	Homogenization method	*In vitro* experiments: DPPH antioxidant assay, MTT cytotoxicity assay, wound scratch assay, antibacterial assay	[155]

Species inclusion criteria:

(1) Anti-aging activity has been verified in peer-reviewed literatures;

(2) Available validation data from in vivo animal models and related mechanistic pathways;

(3) Specific bioactive components have been successfully identified.

Abbreviations

The following abbreviations are used in this Supplementary Materials:

HaCaT cells	Human immortalized keratinocyte cells
GaGs	Glycosaminoglycan
QBL	LarvaQueen Bee Larvae
RJ	Royal Jelly
IDA	Trans-3-indoleacrylic acid
CA	Carminic acid
MAE-P6	Maggot Aqueous Extract，P6
—	Not mentioned
